# The human CTF4-orthologue AND-1 interacts with DNA polymerase α/primase via its unique C-terminal HMG box

**DOI:** 10.1098/rsob.170217

**Published:** 2017-11-22

**Authors:** Mairi L. Kilkenny, Aline C. Simon, Jack Mainwaring, David Wirthensohn, Sandro Holzer, Luca Pellegrini

**Affiliations:** Department of Biochemistry, University of Cambridge, Cambridge CB2 1GA, UK

**Keywords:** DNA replication, replisome, protein–protein interactions, protein–DNA interactions, DNA polymerase, protein hub

## Abstract

A dynamic multi-protein assembly known as the replisome is responsible for DNA synthesis in eukaryotic cells. In yeast, the hub protein Ctf4 bridges DNA helicase and DNA polymerase and recruits factors with roles in metabolic processes coupled to DNA replication. An important question in DNA replication is the extent to which the molecular architecture of the replisome is conserved between yeast and higher eukaryotes. Here, we describe the biochemical basis for the interaction of the human CTF4-orthologue AND-1 with DNA polymerase α (Pol α)/primase, the replicative polymerase that initiates DNA synthesis. AND-1 has maintained the trimeric structure of yeast Ctf4, driven by its conserved SepB domain. However, the primary interaction of AND-1 with Pol α/primase is mediated by its C-terminal HMG box, unique to mammalian AND-1, which binds the B subunit, at the same site targeted by the SV40 T-antigen for viral replication. In addition, we report a novel DNA-binding activity in AND-1, which might promote the correct positioning of Pol α/primase on the lagging-strand template at the replication fork. Our findings provide a biochemical basis for the specific interaction between two critical components of the human replisome, and indicate that important principles of replisome architecture have changed significantly in evolution.

## 1. Introduction

Duplication of the genome before cell division is performed by a multi-protein assembly known as the replisome [1,2]. The replisome contains the necessary enzymatic activities, such as DNA unwinding and nucleotide polymerization, to copy the genetic information using the parental DNA strands as templates. In addition, integral to the replisome assembly are several non-enzymatic components that are important for efficient DNA replication, under normal conditions and in situations of replicative stress. One of the best characterized of these replisome components is the yeast Ctf4 (chromosome transmission fidelity 4), a homotrimeric hub that links the Cdc45-MCM-GINS (CMG) DNA helicase with DNA polymerase α (Pol α)/primase, and interacts with protein factors involved in various DNA metabolic processes, such as the helicase–nuclease Dna2, the sister chromatid cohesion helicase Chl1 and the rDNA compaction protein Tof2 [3–8]. Ctf4 deficiency causes a pleiotropic phenotype, consisting of sensitivity to DNA damaging agents, faulty sister chromatid cohesion and alterations in the rDNA gene locus, which highlights its importance in maintaining genome stability during DNA replication [7,9–12]. Ctf4 orthologues have been identified in various eukaryotic organisms, including fission yeast (Mcl1) [13], *Drosophila* (Ctf4) [14] and humans (acidic and nucleoplasmic DNA-binding protein; AND-1) [15,16], suggesting that its functional role has been conserved throughout evolution.

To understand the molecular mechanisms of DNA synthesis, it will be essential to elucidate in detail the molecular principles of replisome architecture. We have recently made an important advance by explaining how the protein hub Ctf4 interacts with its partners at the replication fork [6,7]. One pertinent question is to what extent the network of contacts holding together the eukaryotic replisome have been conserved in evolution. We decided to explore this question by biochemical and structural characterization of the human CTF4-orthologue AND-1. AND-1 clearly shares some or possibly all the functions of its yeast counterpart in maintaining genome stability and establishing sister chromatid cohesion [15–20]. This is a likely consequence of its sequence similarity with Ctf4 (21% identity and 36% similarity over 722 out of 1129 aligned residues) and their shared domain structure, comprising an N-terminal β-propeller domain and a C-terminal SepB domain, the latter being responsible for trimerization and for interactions with proteins harbouring a Ctf4-interacting peptide (CIP) motif [21,22]. However, at 1129 amino acids, AND-1 is considerably larger than Ctf4 (879 amino acids) and contains an extended C-terminal region, including an HMG-box domain, that is not present in Ctf4.

Here, we show that the SepB domain of AND-1 is a structural orthologue of yeast Ctf4, that it exists in a trimeric form and that it binds to Pol α/primase, like its yeast orthologue Ctf4. However, we find that although the AND-1 SepB domain has retained a weak affinity for the CIP sequence of human Pol α, the principal Pol α-binding region of AND-1 is represented by its C-terminal HMG box. We demonstrate that the HMG box makes a specific contact with the N-terminal domain of Pol α's B subunit, that had been previously demonstrated to mediate Pol α/primase's recruitment by the T-antigen helicase for SV40 replication [23,24]. Furthermore, we identify a novel DNA-binding activity in AND-1 which maps to the intervening region between the SepB domain and the HMG box, and might act to guide the lagging-strand template towards Pol α/primase.

These findings represent an important advance in our understanding of the interaction between human AND-1 and its protein client Pol α/primase in the mammalian replisome, and therefore in the architecture of the eukaryotic replication fork. They further highlight how similarities in ternary and quaternary structure between orthologous DNA replication factors can conceal mechanistic differences in their functional behaviour.

## 2. Results

### 2.1. Human AND-1 interacts with Pol α/primase

**Figure 1.**
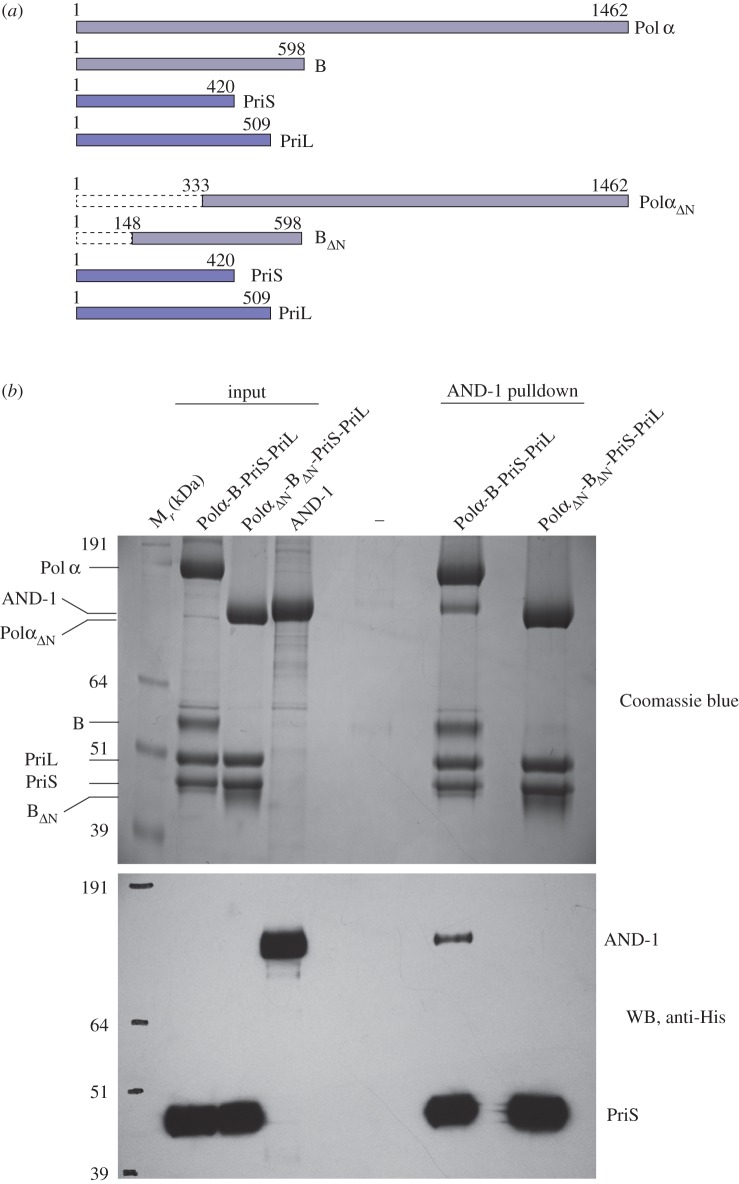
Interaction between human AND-1 and Pol α/primase. (*a*) Subunit structure and construct boundaries of the full-length and truncated versions of the Pol α/primase complex used in this study. (*b*) AND-1 pulldown by full-length and truncated versions of Pol α/primase. In the experiment, double StrepII-tagged Polα-B-PriS-PriL and Polα*_Δ_*_N_-B*_Δ_*_N_-PriS-PriL were tested for interaction with full-length His-myc-AND-1 on Strep-Tactin Superflow resin. (Top panel) Coomassie-stained SDS–PAGE. (Bottom panel) corresponding anti-His western blot (WB). The (–) lane shows AND-1 binding to beads alone (negative control). His-tagged PriS was also detected.

We expressed and purified human AND-1 and Pol α/primase, as well as a truncated version of Pol α/primase lacking the N-terminal regions of Pol α's catalytic subunit and its B subunit (figure 1*a*). Our pulldown experiments with purified components confirmed earlier reports of an interaction between AND-1 and Pol α/primase in cell extracts (figure 1*b*) [15,25]. Our experiments further showed that the interaction is mediated by the N-terminal regions of Pol α and its B subunit, as no binding was observed in the case of the truncated Pol α/primase (figure 1). These results are consistent with our previous findings for the orthologous yeast proteins, where the interaction between Ctf4 and Pol1 (yeast Pol α catalytic subunit) is mediated by the CIP motif of Pol1, present in its N-terminal region [6].

### 2.2. Structure of human AND-1 SepB domain and interaction with Pol α Ctf4-interacting peptide

**Figure 2.**
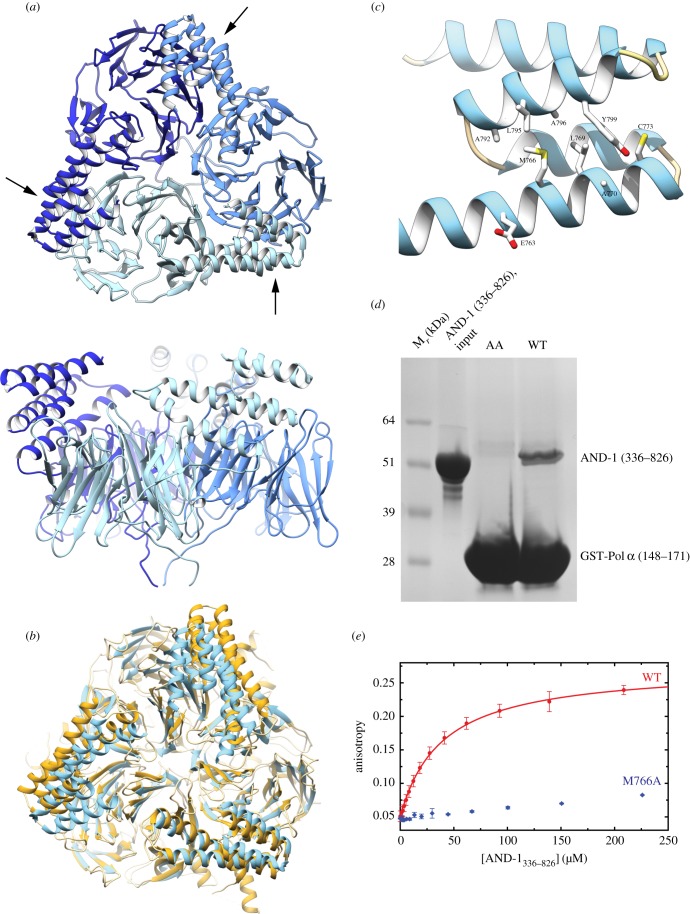
AND-1 trimerization and Pol α CIP binding. (*a*) Top and side views of the crystal structure of the human AND-1 SepB domain. The protein is drawn as ribbons, with each protomer in a different hue of blue. The arrows point to the CIP-binding site of each AND-1 protomer in the trimer. (*b*) Superposition of human AND-1 SepB and Ctf4_CTD_ (PDB ID: 4C8H). The proteins are shown as ribbons, and coloured light blue (AND-1) and yellow (Ctf4). (*c*) The CIP-binding site of human AND-1. The amino acid side chains of residues forming the putative interaction surface in the helical region of the SepB domain are shown as sticks. (*d*) Pulldown experiment of AND-1 SepB (336–826) with WT GST-Pol *α* (148–171) and double-mutant protein (AA), corresponding to alanine mutation of I162 and L163. The results are shown as a Coomassie-stained SDS–PAGE. (*e*) Affinity measurement by fluorescent polarization of fluorescein-labelled Pol α CIP for WT and M766A AND-1 SepB. Each data point is the mean of three independent experiments.

To investigate further the structural basis for the interaction of AND-1 with Pol α, we determined the crystal structure of amino acids 329–826 of human AND-1, comprising its SepB domain, which correspond to the region of Ctf4 (Ctf4_CTD_) that we had previously characterized (figure 2*a*,*b*; electronic supplementary material, table S1) [6]. Despite the low sequence identity (17%, based on structure superposition), the AND-1 SepB structure bears a remarkable resemblance to that of yeast Ctf4_CTD_, with the same tertiary structure comprising a six-bladed β-propeller fused to a C-terminal bundle of five α-helices, and an RMSD value of 1.8 Å over 305 C_α_ positions. In the AND-1 structure, the trimeric arrangement of protomers seen for Ctf4 arises from crystallographic symmetry, and it was confirmed by size-exclusion chromatography multi-angle laser light scattering measurements (electronic supplementary material, figure S1). These findings agree with a crystallographic analysis of human AND-1 that was reported earlier this year [22].

Our data provide strong structural evidence that human AND-1 might behave in the same fashion as yeast Ctf4_CTD_, by acting as a protein hub at the replication fork. Indeed, inspection of the putative binding site for its client proteins, based on structural homology with the Ctf4_CTD_ structure, highlights a solvent-exposed hydrophobic patch formed by M766, A770, A792, L795 and Y799 (figure 2*c*). To test whether AND-1 interacts with its client proteins in the same fashion as Ctf4_CTD_, we performed pulldown experiments of AND-1_336–826_ with a GST-tagged Pol α region spanning residues 148–171, that encompasses its putative AND-1 binding site or CIP (electronic supplementary material, figure S2); we used either the wild-type (WT) sequence or a sequence containing a double alanine mutation of amino acids I162 and L163 (AA) in the middle of the CIP motif. The pulldown experiments showed a clear interaction of Pol α_148–171_ with AND-1_336–826_, which was not observed for the double alanine mutant (figure 2*d*). Together, these findings indicate that AND-1_336–826_ is a structural and oligomeric analogue of Ctf4_CTD_, and that it interacts with its client protein Pol α using the same molecular mechanism.

To obtain a quantitative estimate of the affinity of human Pol α CIP towards AND-1_336–826_, we measured the fluorescence polarization of a fluorescein-labelled Pol *α* CIP sequence spanning amino acids 152-DLSKDGLLGDILQDLNTETP-171, in the presence of increasing amounts of AND-1_329–826_ (figure 2*e*). The alanine mutation of residue M766, at the centre of the putative Pol α CIP-binding site in AND-1 (figure 2*c*; electronic supplementary material, figure S3), was used as control for the specificity of the interaction. The resulting dissociation constant of 39 µM showed that the interaction of AND-1 with Pol *α* CIP is surprisingly weak, and prompted us to ask whether other regions of AND-1 and Pol α/primase contribute to their reciprocal association.

### 2.3. The B subunit of Pol α binds AND-1

**Figure 3.**
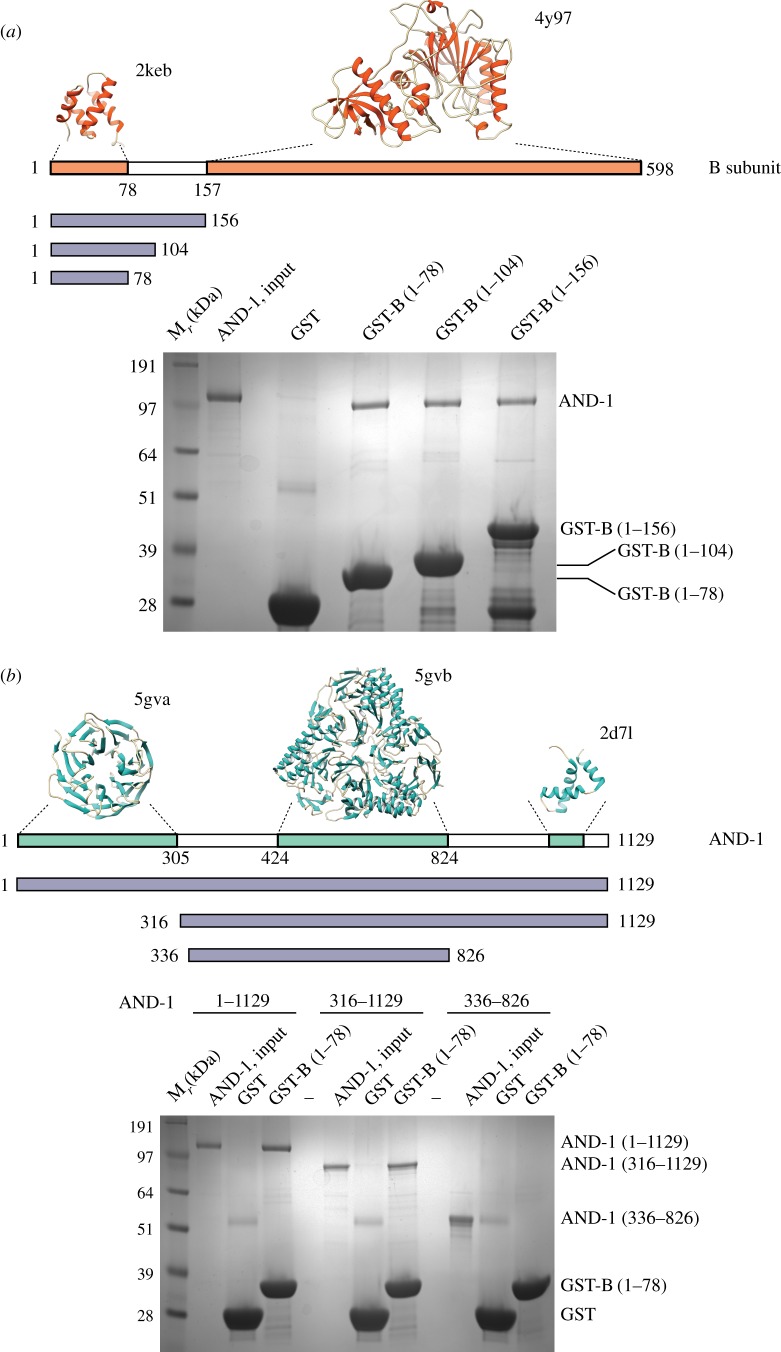
The AND-1_CT_ interacts with the N-terminal domain of the B subunit. (*a*) Pulldown experiment on glutathione agarose of full-length AND-1 with different GST constructs of the B subunit, analysed by SDS–PAGE and Coomassie staining. The domain structure of the B subunit is shown above the experiment, together with the PDB ID of each domain. The boundaries of each B construct tested in the pulldown are also shown. (*b*) Pulldown experiment on glutathione agarose of AND-1 constructs with GST-B_NTD_ (amino acids 1–78), analysed by SDS–PAGE and Coomassie staining. The domain structure of human AND-1 is shown above the experiment, together with the PDB ID of each domain. The boundaries of each AND-1 construct tested in the pulldown experiment are also shown.

As the pulldown experiment of figure 1*b* showed loss of AND-1 binding with a truncated version of Pol α/primase lacking the first 148 amino acids of the B subunit, we sought to determine whether the N-terminal region of the B subunit contributed to the interaction with AND-1. Indeed, pulldown experiments of AND-1 with the N-terminal region of the B subunit revealed a clear interaction, which could be refined further to its first 78 amino acids, known to contain a four-helix bundle domain (B N-terminal domain or B_NTD_) (figure 3*a*) [23]. Reciprocal pulldown experiments aimed at mapping the region of AND-1 responsible for the interaction with the B subunit showed that the binding site resided within AND-1's C-terminal region (AND-1_CT_; amino acids 827–1129) extending beyond its SepB domain (figure 3*b*).

### 2.4. Specific interaction of AND-1 HMG box with the B_NTD_

**Figure 4.**
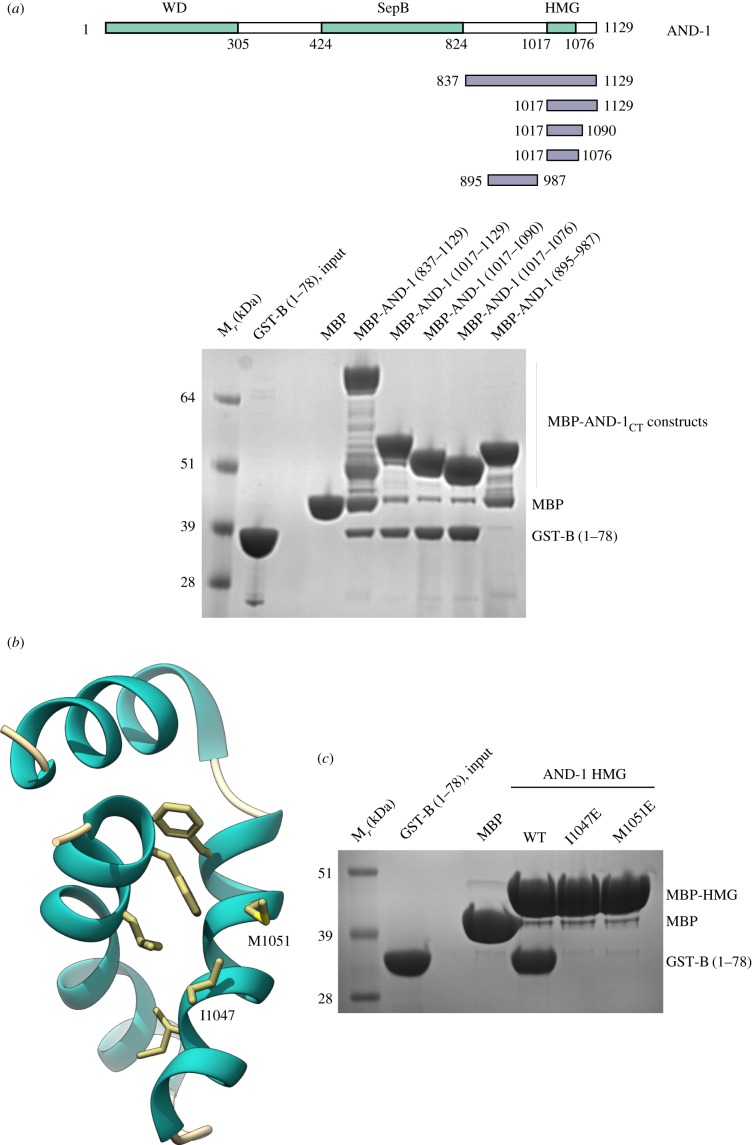
The HMG box of AND-1 mediates the interaction with the B subunit. (*a*) Pulldown experiments on amylose resin of GST-B_NTD_ by MBP-tagged AND-1_CT_ constructs, analysed by SDS–PAGE and Coomassie staining. The boundaries of each AND-1_CT_ construct tested in the experiment are shown above the experiment. (*b*) Structure of the human AND-1 HGM-box domain (PDB ID 2D7L), drawn as ribbon and coloured according to secondary structure, with α-helices in light sea green. Amino acids I1047 and M1051 are part of a solvent-exposed hydrophobic patch on the HMG-box surface, as shown. (*c*) Pulldown experiment on amylose resin of GST-B_NTD_ by MBP-tagged WT and I1047E, M1051E mutant AND-1 HMG-box proteins, analysed by SDS–PAGE and Coomassie staining.

The AND-1_CT_ is predicted to be largely unstructured, except for an HMG-box domain that was mapped to near the C-terminus of the protein [21] (PDB ID 2D7L). Indeed, further experiments showed that the HMG-box sequence was sufficient to bind to B_NTD_, with an affinity that appears to be similar to that possessed by the entire AND-1_CT_ (figure 4*a*). The GST-B_NTD_ and AND-1 HMG proteins co-eluted over size-exclusion chromatography, confirming the interaction (electronic supplementary material, figure S4).

To investigate the specific structural basis for the interaction between AND-1 HMG box and B_NTD_, we generated a panel of structure-based single-point mutants for both proteins, and tested whether the mutations had affected their ability to interact. The mutations were designed to target exposed hydrophobic patches on the surface of each domain, as these typically mediate protein–protein interactions. In the case of AND-1 HMG box, we identified a contiguous cluster of hydrophobic amino acids resulting from the antiparallel packing of the second and third α-helix (figure 4*b*). Introducing point mutations I1047E and M1051E in the HMG box, which reversed the chemical nature of the amino acid, abolished its interaction with B_NTD_ (figure 4*c*; electronic supplementary material, figure S5).

**Figure 5.**
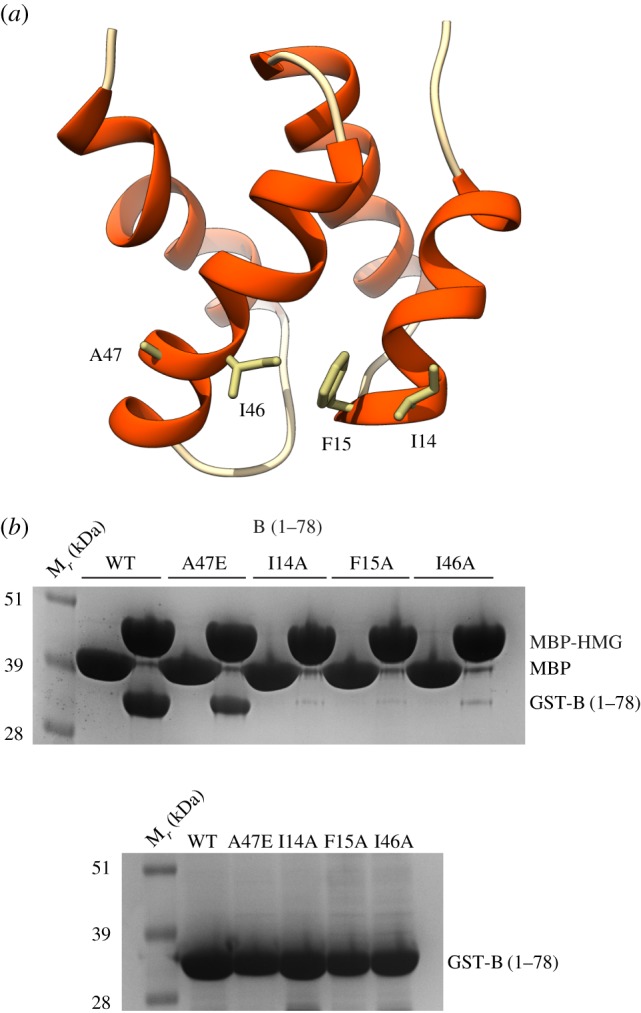
B_NTD_ structure and its binding site for AND-1 HMG box. (*a*) Structure of the human B_NTD_ (PDB ID 2KEB), drawn as ribbon and coloured per secondary structure, with α-helices in red. The side chains of amino acids I14, F15, I46 and A47 form an exposed hydrophobic strip across the B_NTD_ surface. (*b*) Pulldown experiment on amylose resin of WT and single-point I14A, F15A, I46A and A47E mutant GST-B_NTD_ proteins, by MBP-tagged AND-1 HMG box, analysed by SDS–PAGE and Coomassie staining. The bottom panel shows the input amounts of the GST-B_NTD_ proteins.

The B_NTD_ folds in a four-helix bundle domain [23]. Inspection of the available B_NTD_ structure (PDB ID 2KEB) for hydrophobic patches that could complement the one identified in the HMG box structure showed the presence of a contiguous set of hydrophobic residues, I14, F15, I46 and A47, located on the second and third helix of the B_NTD_ structure (figure 5*a*). Alanine mutations of I14, F15 and I46 in the B_NTD_ caused loss of interaction with AND-1 HMG box, and glutamate mutation of the A47 reduced its affinity (figure 5*b*; electronic supplementary material, figure S5). Interestingly, I14 had been shown previously to be important for the interaction of the B subunit with the SV40 T-antigen helicase [23,24].

### 2.5. Comparing Pol α Ctf4-interacting peptide and B_NTD_ affinities for AND-1

**Figure 6.**
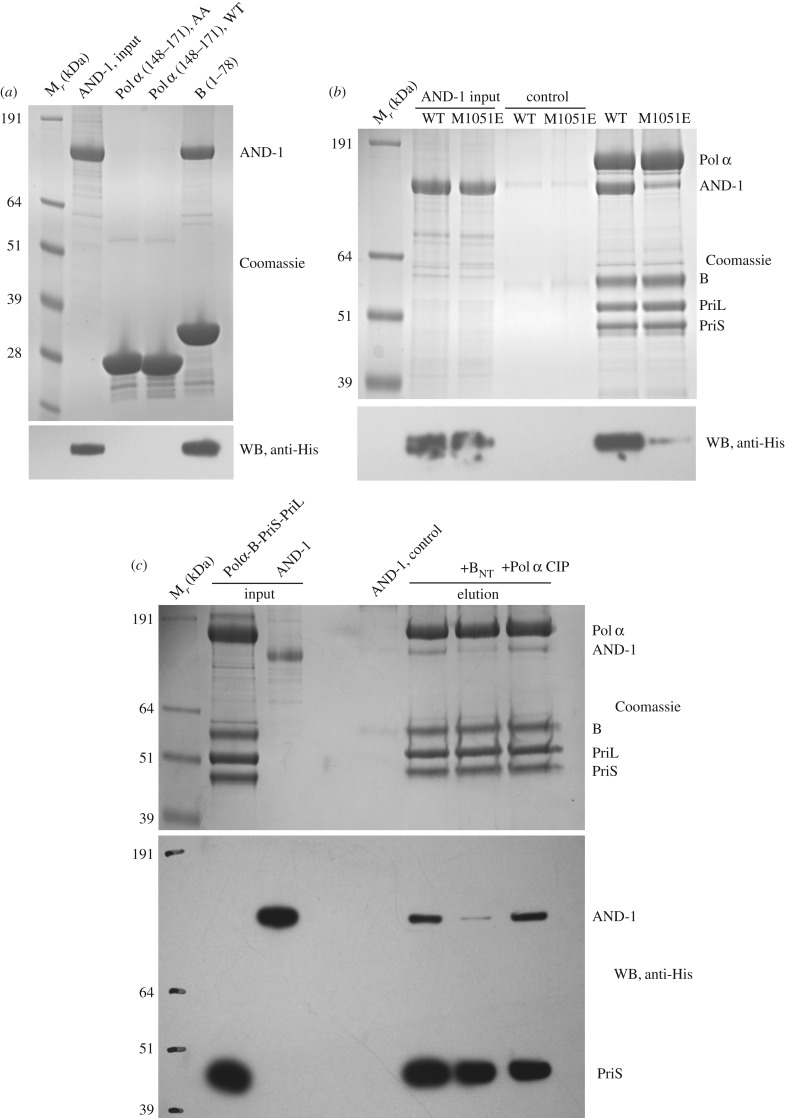
The interaction of AND-1 with the B subunit is stronger than the interaction with Pol α CIP. (*a*) Full-length His-myc-AND-1 pulldown by equimolar amounts of GST-tagged Pol α CIP and B_NTD_ on glutathione agarose. GST-CIP_AA_ was used as negative control (figure 2*d*). (Top panel) Coomassie-stained SDS–PAGE gel. (Bottom panel) Corresponding anti-His western blot (WB). (*b*) Pulldown of WT and M1051E AND-1 by Pol α/primase. StrepII-tagged Pol α/primase was immobilized on Strep-Tactin Superflow resin and released by desthiobiotin. (Top panel) Coomassie-stained SDS–PAGE. (Bottom panel) Corresponding anti-His western blot. (*c*) Competition co-precipitation. Full-length StrepII-tagged Pol α/primase was tested for interaction with full-length His-myc-AND-1 on Strep-Tactin Superflow resin, in the presence of excess purified B_NTD_ or Pol α CIP. AND-1 control comprised resin only with AND-1. (Top panel) Coomassie-stained SDS–PAGE. (Bottom panel) corresponding anti-His western blot (His-tagged PriS is also detected).

The discovery of a new interaction site in AND-1 for Pol α/primase, mediated by the unique C-terminal HMG box present in the human CTF4-orthologue, points to a different, more complex mode of interaction with the DNA polymerase than observed previously for yeast Ctf4. To assess the relative binding strength of AND-1's HMG box and SepB domain with the B subunit and the CIP motif of Pol α, respectively, we performed comparative binding studies with full-length proteins. Collectively, the experiments indicate that the novel interaction mediated by the HMG box is the dominant contact between AND-1 and Pol α/primase (figure 6). First, using the same concentration of full-length AND-1 protein, we could observe AND-1 pulldown by GST-B_NTD_ but not by GST-Pol α CIP (figure 6*a*). Second, introduction of the single-point HMG-box mutation M1051E is sufficient to lose most of the interaction with full-length AND-1, despite the presence of an intact CIP in Pol α/primase (figure 6*b*). Third, competition experiments where the association of full-length AND-1 with Pol α/primase was challenged by addition of free B_NTD_ or free Pol α CIP showed that the B_NTD_, but not Pol α CIP, caused reduced recovery of AND-1 from the resin (figure 6*c*).

### 2.6. AND-1_CT_ interacts with DNA

Being responsible for initiating DNA synthesis at the fork, Pol α/primase is expected to be positioned close to the CMG helicase, poised to prime synthesis on the lagging-strand template. As the protein factor that tethers Pol α/primase to the fork and possibly acts as a bridge between the polymerase and the helicase, AND-1 might be expected to possess an independent DNA-binding activity. This activity would contribute to directing the unwound DNA strand, sterically excluded from the helicase and acting as lagging-strand template, towards the polymerase, based on current models of the yeast replisome [26,27].

**Figure 7.**
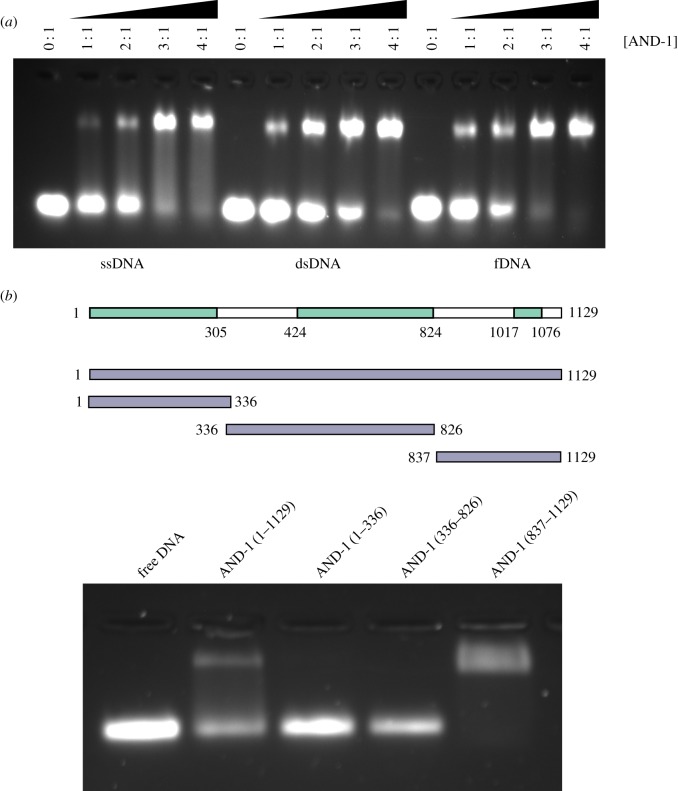
DNA binding of human AND-1. (*a*) EMSA of ssDNA, dsDNA and fDNA, in the presence of increasing amounts of AND-1. (*b*) Domain mapping of the DNA-binding activity of human AND-1. The AND-1 constructs were mixed with ssDNA 40mer in a 2 : 1 stoichiometric ratio. A drawing of the AND-1 constructs tested in the assay is shown above the experiment.

**Figure 8.**
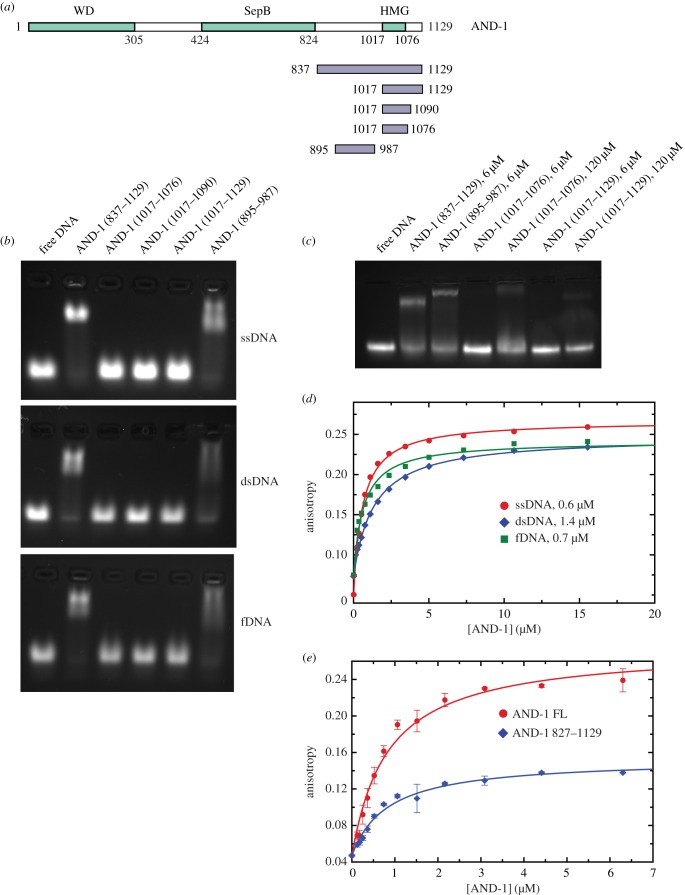
Mapping the DNA-binding region of AND-1_CT_. (*a*) Drawing of the AND-1 CT constructs tested for DNA-binding. (*b*) EMSA of ssDNA, dsDNA and fDNA by different MBP-tagged AND-1_CT_ constructs. (*c*) Repeat of experiment in (*b*), testing binding of an ssDNA 60mer by an excess of HMG-box constructs 1017-076 and 1017-1129. (*d*) Fluorescence anisotropy measurements of AND-1 binding to DNA. DNA-binding of full-length AND-1 to fluorescein-labelled ssDNA, dsDNA and fDNA. The dissociation constant values for AND-1 binding to each DNA substrate are reported in the panel. (*e*) DNA-binding of full-length (FL) AND-1 and AND-1_CT_ with fluorescein-labelled ssDNA 40-mer. Each data point is the mean of three independent experiments.

We tested the ability of AND-1 to bind DNA by electrophoretic mobility shift assay (EMSA) of a range of DNA substrates, including single-stranded (ss) DNA, double-stranded (ds) DNA and fork (f) DNA. We found that AND-1 could form a stable protein–DNA complex with all three substrates, but with higher apparent affinity for ssDNA and fDNA (figure 7*a*). We further found that AND-1_CT_ was the region of AND-1 responsible for DNA binding (figure 7*b*). Mapping the DNA-binding region within the AND-1_CT_ sequence (figure 8*a*; electronic supplementary material, figure S6) showed that, unexpectedly, the HMG box was not involved in DNA binding and that the DNA-binding activity resided within a stretch of approximately 100 amino acids between the C-terminus of the SepB domain and the HMG-box domain (figure 8*b*,*c*). Fluorescence polarization measurements of fluorescein-labelled ssDNA confirmed the ability of AND-1 to bind DNA (figure 8*d*) and that AND-1_CT_ interacted with ssDNA with the same affinity (*K*_d_ approximately 0.8 µM) as the full-length protein (figure 8*e*).

## 3. Discussion

**Figure 9.**
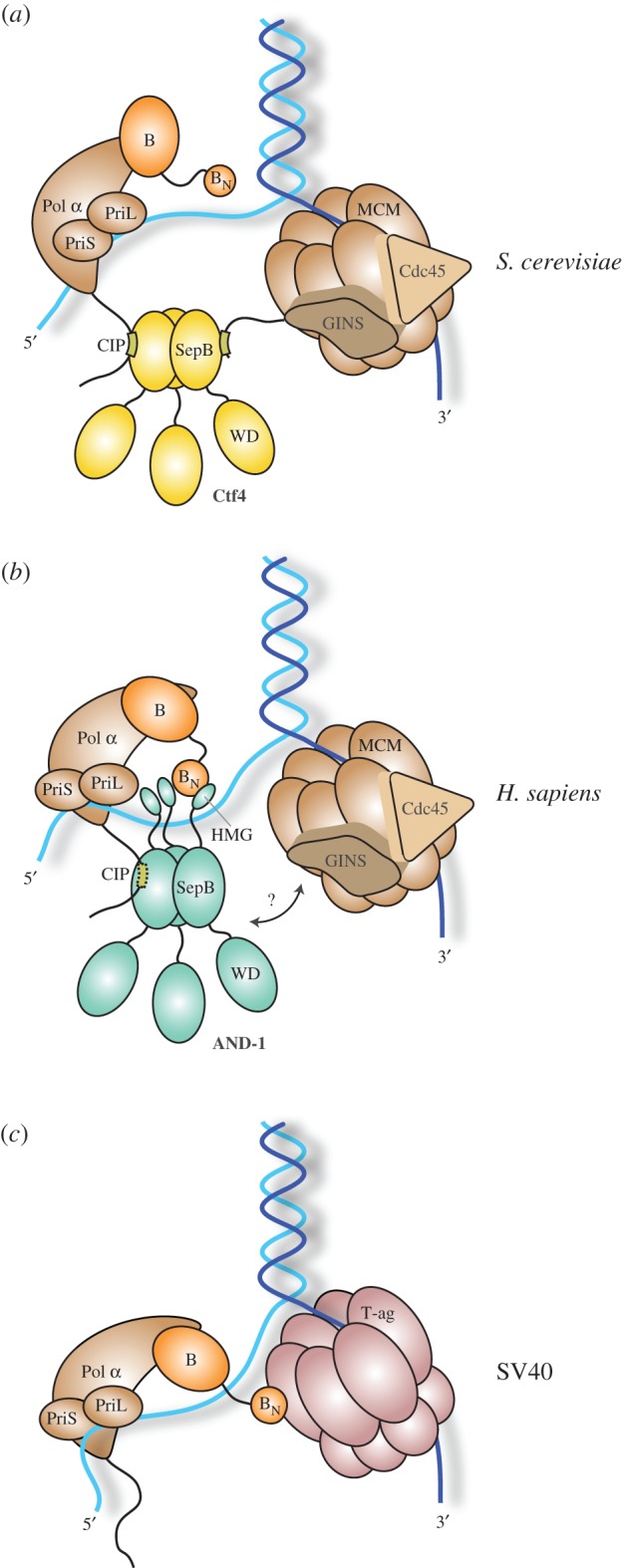
A model for the role of yeast Ctf4 (*a*), and human AND-1 (*b*) at the replication fork, illustrating the known interactions of Ctf4/AND-1 with Pol α/primase and the CMG helicase. Other known interactors of Ctf4, such as the Chl1 helicase, the Dna2 helicase/nuclease and Tof2 are not shown. Whereas recruitment of Pol α/primase to the fork depends on Ctf4 in yeast, human AND-1 interacts with Pol α/primase predominantly via its HMG-box domain. The mode of AND-1 binding to the CMG helicase is still unknown. A model of Pol α/primase recruitment by the T-antigen in SV40 replication is also shown (*c*).

Here, we have described an investigation into the interaction of AND-1, the human orthologue of the yeast replisome factor Ctf4, with DNA polymerase α/primase. We have found that, although AND-1 does interact with Pol α via the evolutionarily conserved CIP motif of the polymerase, binding is dominated by a specific interaction of AND-1's C-terminal region with the B subunit of Pol *α*. The large C-terminal sequence after the conserved SepB domain is a unique feature of mammalian AND-1, as it is absent in yeast Ctf4. Although predicted to be mostly disordered, AND-1_CT_ contains a previously identified HMG-box domain [21]. We show that the HMG box is responsible for the interaction with the B subunit of Pol α/primase and identify specific hydrophobic amino acids on its surface that mediate the interaction. These findings are summarized in figure 9.

AND-1 binding by Pol α/primase is dependent on a small helical domain in the N-terminus of the B subunit. The role of the B subunit in DNA replication had remained poorly understood, although it is encoded by an essential gene in budding yeast [28]. Here, we have shown that one role of the B subunit is to drive the association with AND-1 and therefore presumably with the rest of the human replisome. Interestingly, and in agreement with a role as a protein–protein interaction module reported here, the equivalent domain in the B subunit of Pol *ɛ* mediates an important interaction with the Psf1 subunit of the GINS complex [29].

Intriguingly, the AND-1 binding site on the surface of the B_NTD_ overlaps with the previously identified site that is targeted by the T-antigen helicase for recruitment of Pol α/primase during SV40 replication [23] (figure 9). Given the need to hijack the polymerase for its own replication, it would make sense for the virus to target the main mode of association of Pol α/primase with the replisome. Thus, the observation of a shared B_NTD_ interface for AND-1 and T-antigen corroborates our finding that the interaction of the B subunit with the HMG box is the primary means of AND-1-dependent recruitment of Pol α/primase to the replisome.

In addition to interacting with Pol α/primase, AND-1_CT_ can bind ssDNA, thus pointing to a potential role for AND-1 in directing the lagging-strand template towards Pol α/primase for priming DNA synthesis. When combined, these observations suggest that AND-1 mediates Pol α/primase recruitment to the fork and contributes to the geometry of the interaction of Pol α/primase with the DNA template. Our observation of a DNA-binding activity in AND-1 agrees with previous data [21,22]. However, we find that DNA binding by AND-1_CT_ is not mediated by the HMG, as previously reported [21,22], but by the intervening region between the SepB and HMG domains.

Our emerging model of yeast Ctf4 function indicates that it acts as a protein hub at the replication fork, mediating multiple interactions with other replication factors via its Ctf4_CTD_ domain [7]. These interactions help bridge DNA helicase and DNA polymerase, as well as recruiting other proteins with roles in processes associated with DNA replication. The amino acid sequence of human AND-1 shows that it is clearly related to Ctf4, with which it shares its domain structure, formed by an N-terminal WD domain and a C-terminal SepB domain, as well as oligomerization state. Functional interactions with other replication factors such as Timeless, Tipin, Claspin and MCM10 have also been reported [15,25,30]. Thus, the evidence points to a role for AND-1 as a hub for recruitment of client proteins to the fork, in a similar fashion to Ctf4. However, our findings provide evidence for clear mechanistic differences too, concerning the mode of binding to Pol α/primase. The canonical interaction with the Pol α CIP, although detectable, is rather weak, whereas a novel, stronger contact is observed with the B subunit, which depends on the HMG-box domain in the AND-1_CT_, not present in Ctf4. Furthermore, the ability of AND-1_CT_ to interact with ssDNA, at a site juxtaposed to the location of the HMG box, suggests that AND-1 helps position Pol α/primase at the fork via both protein–protein and protein–DNA contacts.

What do these biochemical observations tell us about the architecture of the human replisome? Although it is impossible to draw reliable conclusions in the absence of structural information, these data point to a distinct reciprocal arrangement of AND-1 and Pol α/primase at the replication fork (figure 9). The picture is further complicated by our ignorance concerning how AND-1 is linked to the human CMG helicase, as its GINS Sld5 subunit of the human helicase lacks the CIP motif of its yeast counterpart. More biochemical and structural investigations will be required to shed more light on the architecture of the human replisome.

## 4. Material and methods

### 4.1. Protein cloning, expression and purification

Double StrepII-tagged human DNA polymerase α (1–1462) and its B subunit (1–598) were cloned into the pFBDM vector. The human primase subunits, His_10_-PriS (1–420) and PriL (1–509), were similarly cloned into a separate pFBDM vector. These vectors were each used to generate a recombinant baculovirus using the MultiBac system [31]. Expression of the heterotetrameric Pol α/primase complex entailed co-infection of Sf9 insect cells (at a density of 2 × 10^6^ cells ml^−1^) with both baculoviruses, after which the cells were incubated for 72 h at 27°C, with shaking at 120 rpm. An N-terminally truncated version of the complex, comprising double StrepII-tagged Pol α (334–1462) and B subunit (149–598), was cloned and expressed with full-length primase in the same way. Full-length and truncated Pol α/primase were purified using Strep-Tactin Superflow resin (IBA), and confirmed DNA-free by UV absorption spectroscopy.

Synthetic, annealed oligonucleotides (Sigma Aldrich) were used to clone the Pol α CIP sequence 148-DKAVDLSKDGLLGDILQDLNTETP-171, and a double alanine mutant 148-DKAVDLSKDGLLGDAAQDLNTETP-171 into vector pGAT2 [32]. Proteins were expressed in Rosetta2(DE3) cells (Novagen), and purified using Ni-NTA agarose (Qiagen).

Full-length His_10_-myc-tagged AND-1 (1–1129) was cloned into the pFBDM vector, which was subsequently used to generate recombinant baculovirus using the MultiBac system [31]. AND-1 protein was expressed by infecting Sf9 insect cells (density = 2 × 10^6^ cells ml^−1^) with the baculovirus and shaking the cells at 120 rpm for 72 h at 27°C. Purification involved successive Ni-NTA agarose (Qiagen) and Q-Sepharose anion-exchange (GE Healthcare) chromatography steps. The full-length His_10_-myc-tagged AND-1 M1051E point mutant was cloned, expressed and purified in the same way. Following purification, proteins were confirmed DNA-free by UV absorption spectroscopy.

His_6_-tagged AND-1 SepB (336–826) and AND-1 SepB + CT (316–1129) were individually cloned into pRSF-Duet1 (Novagen) and expressed in Rosetta2(DE3) cells (Novagen). The AND-1 M766A point mutation was introduced by site-directed mutagenesis. Proteins were purified using Ni-NTA agarose (Qiagen) and Q-Sepharose anion-exchange chromatography (GE Healthcare), followed by overnight TEV protease cleavage of the His_6_-tag. Finally, size-exclusion chromatography was performed using a HiLoad 16/60 Superdex 200 prep grade column (GE Healthcare), equilibrated in 25 mM HEPES pH 7.2, 280 mM NaCl and 5 mM DTT. The size-exclusion chromatography confirmed that the M766A point mutant was correctly folded.

AND-1 (1–334) was cloned into the pGAT3 vector and expressed in Rosetta2(DE3) cells (Novagen) [32]. The protein was purified using Ni-NTA agarose (Qiagen) followed by size-exclusion chromatography over a HiLoad 16/60 Superdex 200 prep grade column (GE Healthcare) in buffer containing 25 mM HEPES pH 7.2, 300 mM KCl, 5% (w/v) glycerol.

AND-1 C-terminal constructs (837–1129, 1017–1129, 1017–1076, 1017–1090, 895–987) were cloned into the pMAT11 vector for expression as His_6_-MBP-tagged proteins [32]. Point mutations I1047E and M1051E were introduced into the AND-1(1017–1076) construct, encoding the HMG box, by site-directed mutagenesis. His_6_-MBP-tagged proteins were expressed in Rosetta2(DE3) cells (Novagen), and purified using Ni-NTA agarose (Qiagen) followed by size-exclusion chromatography using a HiLoad 16/60 Superdex 200 prep grade column (GE Healthcare) in buffer containing 25 mM Tris–HCl pH 7.9, 300 mM KCl and 1 mM TCEP. The point mutants were confirmed folded by cleaving the His_6_-MBP tag overnight with TEV protease, followed by Ni-NTA agarose recapture of the His_6_-MBP tag and subsequent size-exclusion chromatography of the AND-1 HMG constructs using a Superdex 75 10/300 GL column (GE Healthcare) in PBS buffer. Untagged AND-1 (837–1129) protein, AND-1_CT_, was prepared by incubating the His_6_-MBP-tagged protein with TEV protease overnight, followed by purification over Ni-NTA agarose (Qiagen) and a Heparin HiTrap 5 ml column (GE Healthcare).

B subunit N-terminal constructs (1–78, 1–104 and 1–156) were cloned into the pGAT2 vector [32]. Point mutations (I14A, F15A, I46A, A47E) were introduced into the B (1–78; B_NT_) construct by site-directed mutagenesis. His_6_-GST-tagged proteins were expressed in Rosetta2(DE3) cells (Novagen), and purified using Ni-NTA agarose (Qiagen). These mutants were confirmed folded by cleaving the His_6_-GST tag overnight with thrombin protease (50 units, Sigma Aldrich), followed by Ni-NTA recapture of the His_6_-GST tag and subsequent size-exclusion chromatography of the B_NTD_ on a Superdex 75 10/300 GL column (GE Healthcare) in PBS buffer.

### 4.2. AND-1 SepB crystallization and structure determination

An AND-1 construct spanning amino acids 329–826 (SepB domain) was cloned into a PRSFDuet-1 vector and transformed in *Escherichia coli* BL21Rosetta2(DE3) (Novagen) for expression. About 3–5 l of bacteria was grown in 2 × YT medium in an orbital shaker at 37°C and 210 rpm until they reached an optical density at 600 nm (OD600) of 0.9. Protein expression was induced with the addition of 0.5 mM IPTG and the cultures were grown for an additional 16 h at 20°C. Bacteria were harvested by centrifugation at 4000*g*, resuspended in 20 mM HEPES pH 7.0, 500 mM NaCl, 10 mM imidazole and sonicated. AND-1 (329–826) was initially purified by nickel affinity chromatography, followed by TEV cleavage of the His-tag, and further purification by anion-exchange chromatography over a 6 ml-RESOURCE Q column (GE Healthcare) and gel-filtration chromatography over a Superdex S200 16/60 column (GE Healthcare) in 25 mM HEPES pH 7.0, 200 mM NaCl, 10% (w/v) glycerol. Peak fractions were pooled, concentrated to 9 mg ml^−1^, supplemented with 1 mM TCEP, flash frozen in liquid nitrogen and stored in small aliquots at −80°C.

Crystals of AND-1 329–826 were grown at 9 mg ml^−1^ using the hanging-drop vapour diffusion technique, in 0.1 MHEPES pH 7.0, 1.1 M di-sodium malonate and 0.5% (v/v) Jeffamine ED-2003, supplemented with 0.36–0.42 M sulfobetaine NDSB-195. For data collection, crystals were cryo-protected using 25% (w/v) glycerol and flash frozen in liquid nitrogen. X-ray diffraction data were collected at beamline I03 of the Diamond Light Source, Oxford, UK, and the data were integrated using XDS [33]. Space group symmetry was assigned in POINTLESS and intensities scaled in AIMLESS [34]. The protein crystallized in the cubic space group F 4 3 2 with unit cell dimensions of *a* = *b* = *c* = 249.72 Å and one AND-1 protomer per asymmetric unit. The structure was solved using PHENIX MR-Rosetta [35] in combination with a Robetta generated fragment library [36] using the Ctf4_CTD_ structure as search model. Local and global protein homology assisted alignments were calculated using the HHpred server [37] and an initial model was generated using PHENIX AutoBuild as part of MR-Rosetta [38]. The crystallographic model was extended and completed by repeated cycles of manual building in Coot and crystallographic refinement with PHENIX Refine [38,39]. The final model was refined using data to 2.5 Å, to R-work and R-free values of 0.173 and 0.210 and a Molprobity score of 1.16 [40]. Amino acids 329–420 and 824–826 were not included in the final model due to missing or poor electron density and are presumed to be disordered.

### 4.3. Pulldown experiments

All experiments were performed in PD buffer (PBS, 5% (w/v) glycerol, 0.5 mM TCEP, 0.2% Igepal), and all proteins were buffer exchanged into this buffer prior to the experiment. BSA (Sigma Aldrich) was present at a concentration of 2.5 mg ml^−1^ in all cases. The same basic procedure was followed for each experiment: saturating quantities of bait protein were bound to 100 µl resin for 30 min at 4°C. The resin was washed with 2 × 1 ml PD buffer, followed by addition of the prey protein. Following a 90 min incubation at 4°C, the resin was washed with 4 × 1 ml PD buffer and the bound proteins eluted and analysed by SDS–PAGE with Coomassie staining. AND-1 was visualized by western blotting using a mouse monoclonal anti-His antibody (H1029, Sigma Aldrich) and HRP-conjugated anti-mouse antibody (W402B, Promega).

For pulldown of full-length AND-1 (WT or M1051E) by Pol α/primase, purified Pol α/primase was bound to 100 µl Strep-Tactin Superflow resin (IBA). About 2.4 nmol purified AND-1 was added, and elution performed with 100 µl PD buffer supplemented with 10 mM desthiobiotin (Sigma Aldrich). GST-Polα fusion proteins were bound to 100 µl glutathione agarose resin (Cube Biotech), after which 20 nmol of purified AND-1 SepB was added, and elution performed with 100 µl PD buffer supplemented with 20 mM reduced glutathione (Sigma Aldrich). GST-B fusion proteins were bound to 100 µl glutathione agarose resin (Cube Biotech), after which 2.2 nmol purified AND-1 (1–1129, 316–1129 or 336–826) was added, and elution performed as described above. MBP-AND-1 constructs were bound to 100 µl amylose resin (NEB), after which 25 nmol purified GST-B was added. Elution was performed with 100 µl PD buffer supplemented with 20 mM maltose (Sigma Aldrich).

For the competition experiment, purified Pol α/primase was bound to 100 µl Strep-Tactin Superflow resin (IBA) and 1 nmol of purified AND-1 was added, together with either 100 nmol B (1–78) or Pol α CIP sequence 156-DGLLGDILQDLNTET-170 (Genosphere Biotechnologies). Elution was performed with 100 µl PD buffer supplemented with 10 mM desthiobiotin (Sigma Aldrich).

### 4.4. DNA constructs

DNA oligonucleotides were purchased from Sigma Aldrich (oligo A: 6FAM-CTTCCGAGACCTTGCCCATCCCGTAGAACCTGTTATCCAA, oligo B: TTGGATAACAGGTTCTACGGGATGGGCAAGGTCTCGGAAG, oligo C: GCTACCTTTGAACCTACGATGATGGGCAAGGTCTCGGAAG). Oligo A was used as the ssDNA substrate, while B or C was annealed to A to generate the dsDNA and fDNA substrates, respectively. An ssDNA 60mer was used in figure 8*c*, of sequence: 6FAM-ATGGTGTGTGTAGGTTAATGTGAGGAGGAGAGGTGAAGAAGGAGGAGAGAAGAAGGAGGC.

### 4.5. Electrophoretic mobility shift assay

All proteins were buffer exchanged into EMSA buffer (25 mM HEPES pH 7.2, 200 mM KCl, 1 mM TCEP) using an Illustra NAP-5 column (GE Healthcare). Sample reactions contained 3 µM 6FAM-labelled DNA (listed above) and the indicated amount of protein. The salt concentration was adjusted to 100 mM KCl and 5 mM MgCl_2_, in a final volume of 20 µl. Reaction mixtures were run for 60 min at 4°C on 0.75% (w/v) agarose gels, at 45 V in 0.5× Tris-borate buffer pH 8.3, in an EM100 gel unit (Cambridge Electrophoresis Ltd) and the gels were visualized under UV light.

### 4.6. Fluorescence polarization

Binding experiments were performed in 96-well plate format. Fluorescence anisotropy measurements were recorded at 25°C in a PHERAstar Plus multi-detection plate reader (BMG Labtech) equipped with fluorescence polarization optic module (*λ*_ex_ = 485 nm; *λ*_em_ = 520 nm). Each data point is the mean of 200 flashes per well, and the voltage gain was set by adjusting the target mP values of fluorescein-labelled peptide or DNA relative to that of fluorescein (35 mP). Curve-fitting was performed in pro Fit 6.1.11 (Quantum Soft) using a Levenberg–Marquardt fitting algorithm.

The optimal concentration of peptide or DNA was determined by calibration curves. To analyse the interaction between Pol α and AND-1 SepB, a fluorescein-labelled Pol α CIP sequence (fluorescein-labelled DLSKDGLLGDILQDLNTETP) was synthesized by Genosphere Biotechnologies. Each well contained 30 nM peptide, in buffer comprising 25 mM HEPES pH 7.2, 140 mM NaCl, 5 mM DTT, 5 mM MgCl_2_ and 5% glycerol. AND-1 SepB was titrated in increasing concentration.

To measure the binding of AND-1 to DNA, fluorescein-labelled ssDNA, dsDNA or fDNA was used (sequences listed above). Each well contained 20 nM DNA in buffer containing 25 mM HEPES pH 7.2, 100 mM KCl and 0.5 mM TCEP. Full-length His_10_-myc-AND-1 (1–1129) or untagged AND-1_CT_ (837–1129) was titrated in increasing concentrations.

### 4.7. Size-exclusion chromatography

To analyse the interaction between AND-1 HMG and B_NTD_, a 250 µl sample containing 42 nmol GST-B(1–78) and 84 nmol AND-1 HMG was injected onto a Superdex 200 10/300 GL column (GE Healthcare) pre-equilibrated in 25 mM HEPES pH 7.2, 100 mM KCl and 0.5 mM TCEP. The individual proteins alone were also analysed. Eluted fractions were analysed by SDS–PAGE with Coomassie staining.

### 4.8. Size-exclusion chromatography and multi-angle laser light scattering

Size-exclusion chromatography experiments in combination with multi-angle laser light scattering were performed using 100 µl of AND-1 336–826 protein at 2–4 mg ml^−1^. The protein was injected onto a Superdex S200 10/300 GL gel-filtration column (GE Healthcare) in 25 mM HEPES pH 7.0, 200 mM NaCl and at a flow rate of 0.5 ml min^−1^. The column was controlled using an Äkta Purifier System (GE Healthcare) and was linked to a DAWN 8^+^ 8-angle light scattering detector (Wyatt Technology) with a fused silica sample cell using a laser wavelength of 664 nm. The change in the refractive index was detected using an Optilab TrEX refractometer with extended range (Wyatt Technology) at a wavelength of 658 nm. Data collection and analysis was carried out using the ASTRA6 software package (Wyatt Technology). Molecular weight determination across the sample peak was carried out using a Zimm-plot derived global fitting algorithm with a fit degree of 1 and a d*n*/d*c* value of 0.1850 ml g^−1^.

## Supplementary Material

Supplementary material

## Data accessibility

The coordinates and structure factors for AND-1 SepB domain have been deposited in the Protein DataBank under accession code 5OGS.
