# Supplementary material for: The human CTF4-orthologue AND-1 interacts with DNA polymerase α/primase via its unique C-terminal HMG box
Source: Open Biol. 2017 Nov 22;7(11):170217. doi: 10.1098/rsob.170217 (PMC5717350; doi:10.1098/rsob.170217)
Supplement: Supplementary material [file rsob170217supp1.pdf]

The human CTF4-orthologue AND-1 interacts with DNA polymerase α/primase via its unique C-terminal HMG box

Mairi L. Kilkenny, Aline C. Simon, Jack Mainwaring, David Wirthensohn, Sandro Holzer, Luca Pellegrini

List of supplementary items:

- Supplementary Table 1
- Supplementary figures 1 to 6

Supplementary table 1. Data collection and refinement statistics.

	AND-1 SepB
**Wavelength (Å)**	0.97625
**Resolution range (Å)**	44.14 - 2.503 (2.593 - 2.503)
**Space group**	F 4 3 2
**Unit cell**	249.7 249.7 249.7 90 90 90
**Total reflections**	395220 (32856)
**Unique reflections**	23558 (2288)
**Multiplicity**	16.8 (14.4)
**Completeness (%)**	99.89 (99.52)
**Mean I/sigma(I)**	24.30 (1.92)
**Wilson B-factor**	49.66
**R-merge**	0.1235 (1.494)
**R-meas**	0.1274 (1.55)
**R-pim**	0.03082 (0.4044)
**CC1/2**	0.999 (0.563)
**Reflections used in refinement**	23555 (2288)
**Reflections used for R-free**	1176 (122)
**R-work**	0.1764 (0.3055)
**R-free**	0.2110 (0.3570)
**CC(work)**	0.966 (0.718)
**CC(free)**	0.856 (0.659)
**Number of non-hydrogen atoms**	3401
**macromolecules**	3221
**ligands**	14
**solvent**	166
**Protein residues**	403
**RMS(bonds)**	0.002
**RMS(angles)**	0.42
**Ramachandran favored (%)**	98.00
**Ramachandran allowed (%)**	2.00
**Ramachandran outliers (%)**	0.00
**Rotamer outliers (%)**	0.57
**Clashscore**	2.96
**Average B-factor**	55.04
**macromolecules**	54.92
**ligands**	82.12
**solvent**	55.13

Statistics for the highest-resolution shell are shown in parentheses.

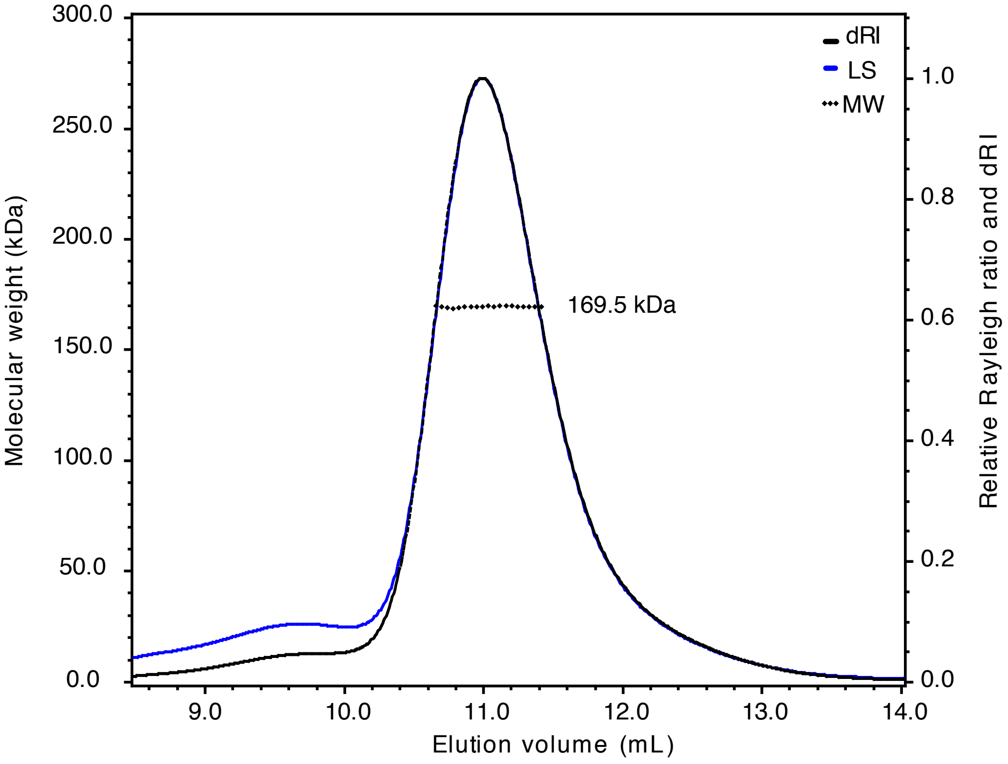

Supplementary figure S1

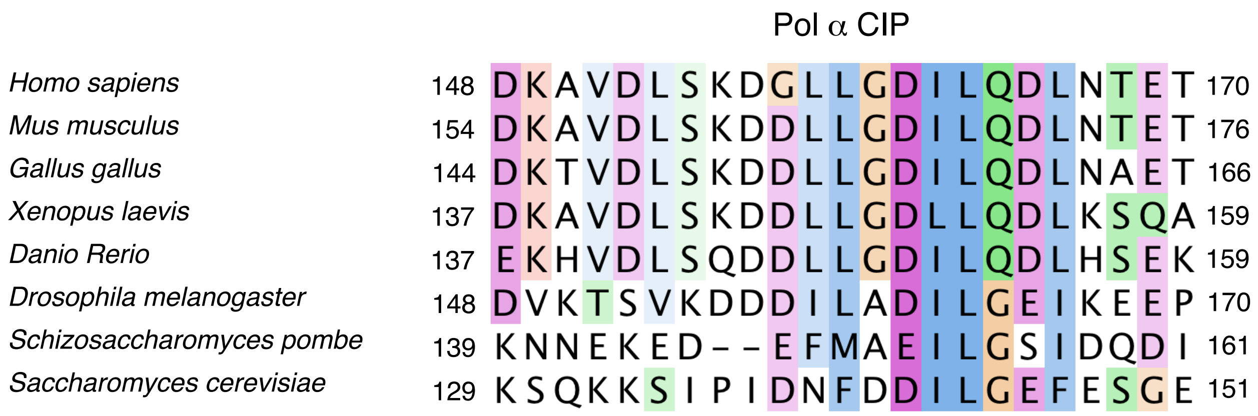

Supplementary figure S2

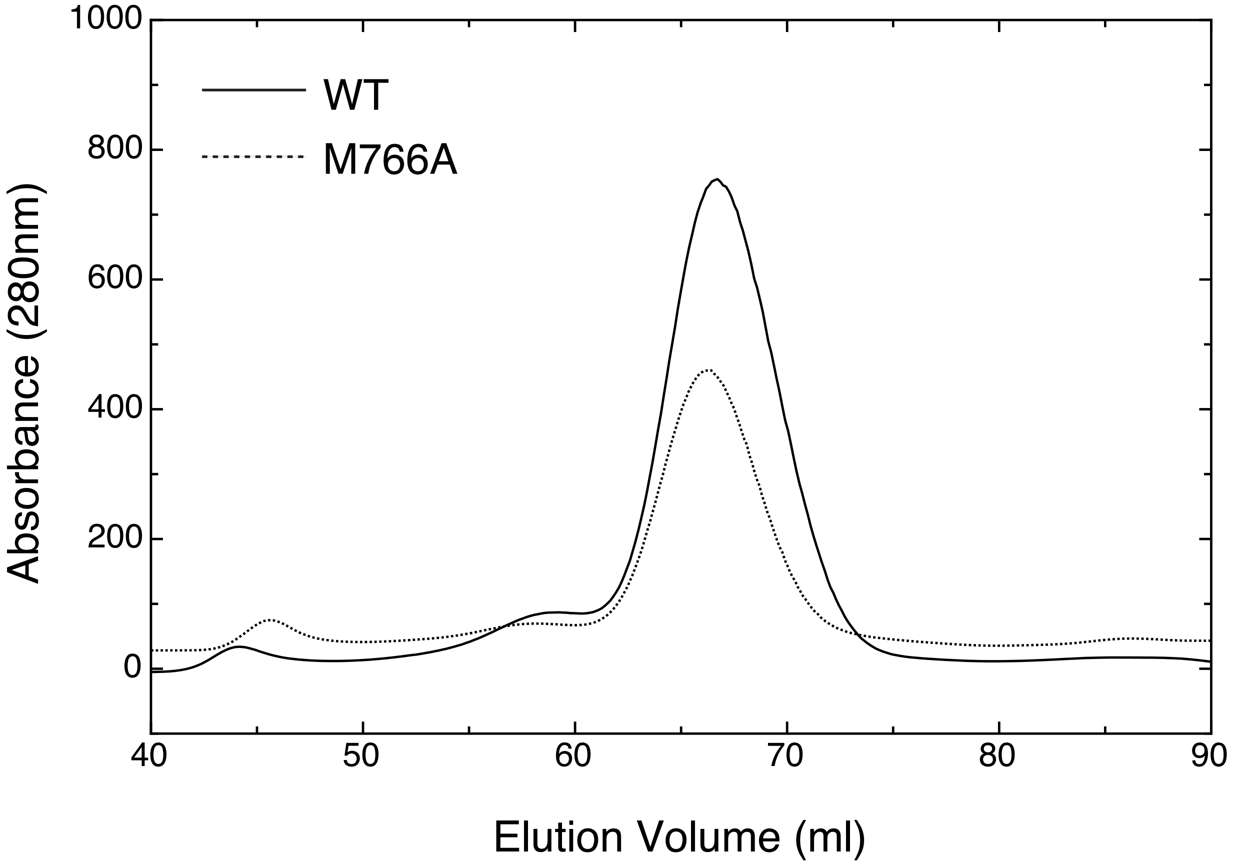

Supplementary figure S3

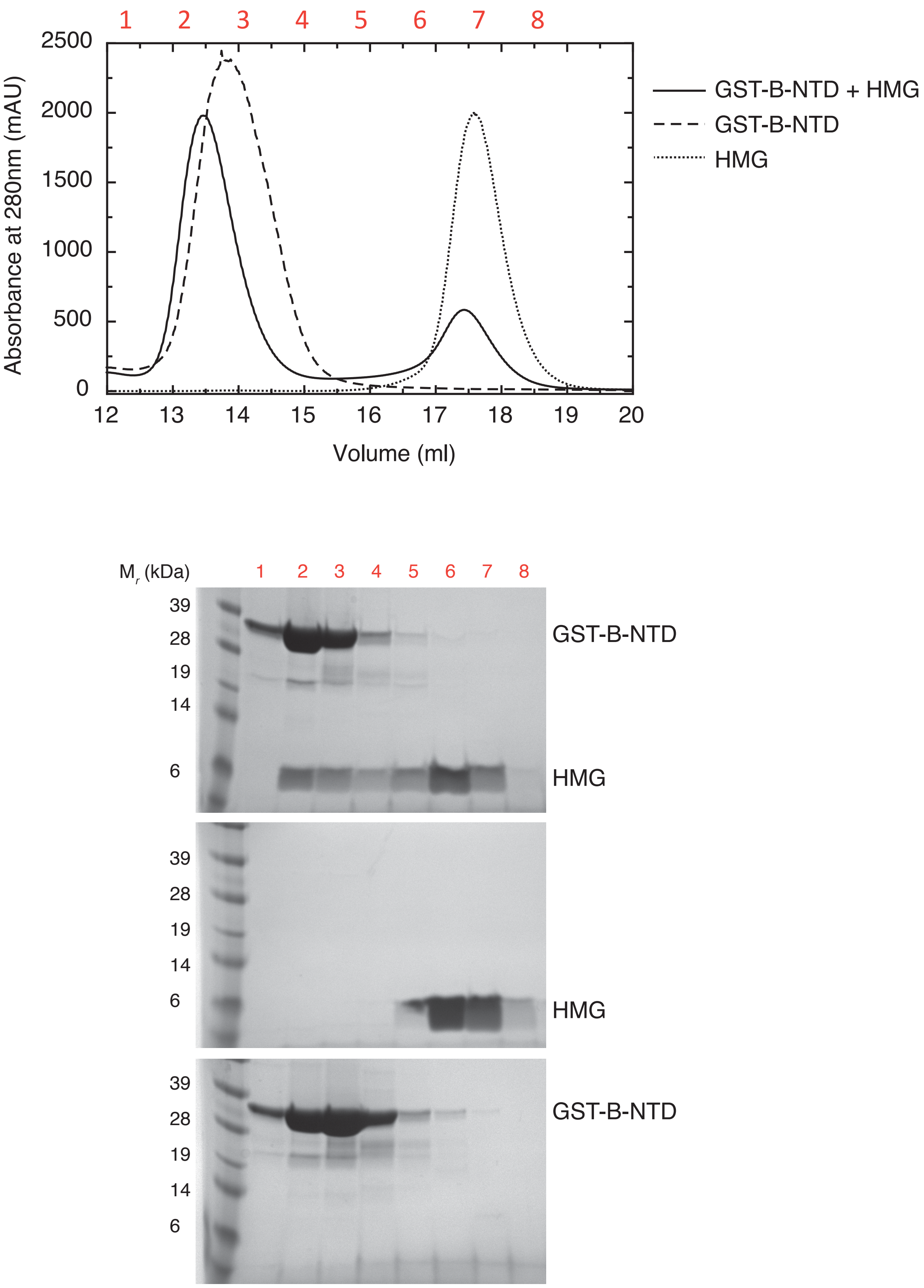

Supplementary figure S4

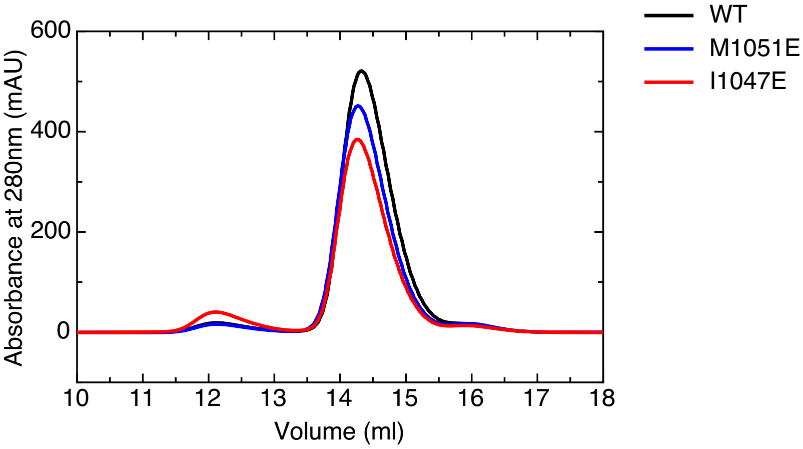

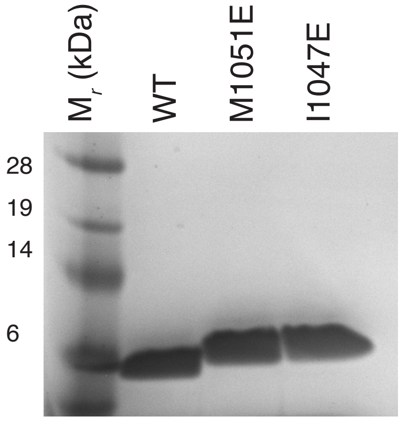

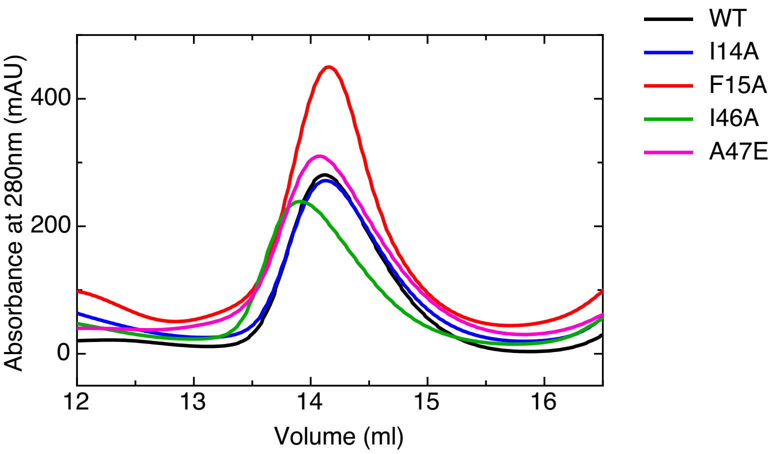

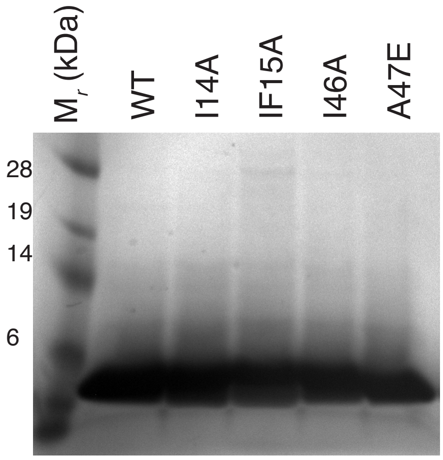

Supplementary figure S5

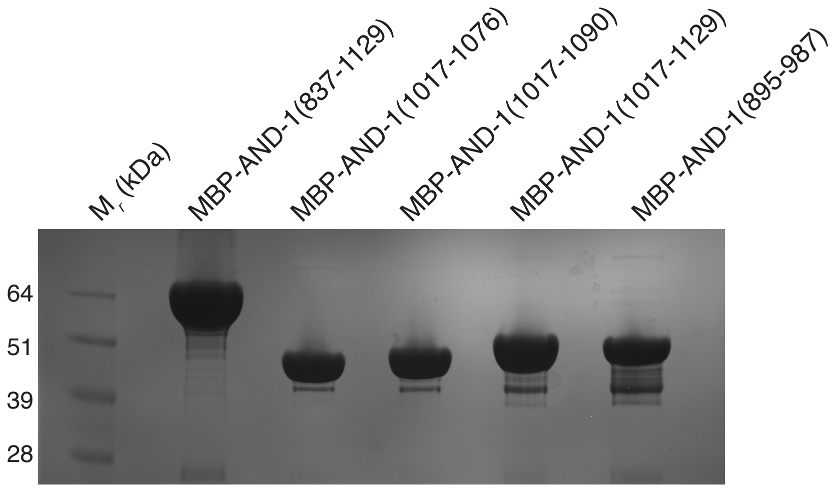

Supplementary figure S6

SUPPLEMENTARY FIGURE LEGENDS

Supplementary figure S1. Size-exclusion chromatography multi-angle laser light scattering analysis of AND-1 336-826. The light scattering (LS) elution trace is coloured in black, the corresponding refractive index change (dRI) is highlighted in blue. The molecular weight calculated across the peak is shown and stated next to the trace.

Supplementary figure S2. Multiple sequence alignment of Pol α CIP sequences. Conserved amino acids are coloured according to their chemical nature.

Supplementary figure S3. Size-exclusion chromatography analysis of purified wild-type and M766A mutant AND-1 SepB domain proteins.

Supplementary figure S4. Reconstitution of the GST-B_{NTD} - AND-1 HMG box complex by size-exclusion chromatography. The top panel shows the chromatographic profiles of the GST-B_{NTD}, AND-1 HMG box and their complex. The bottom panels show the SDS-PAGE analysis of the eluted fractions, stained by Coomassie blue.

Supplementary figure S5. Size-exclusion chromatography and related SDS-PAGE analysis of purified wild-type and mutant AND-1 HMG (top panels) and GST-B_{NTD} proteins (bottom panels).

Supplementary figure S6. Purified MBP-tagged AND-1 proteins used in the DNA-binding assays, analysed by SDS-PAGE and stained with Coomassie.
